# Architecting data-driven microbial electrochemistry from scratch

**DOI:** 10.1016/j.patter.2022.100637

**Published:** 2022-11-11

**Authors:** Waheed Miran, Gaku Imamura, Akihiro Okamoto

**Affiliations:** 1 International Center for Materials Nanoarchitectonics, National Institute for Materials Science, 1-1 Namiki, Tsubaka, Ibaraki 305-0044, Japan; 2 School of Chemical and Materials Engineering, National University of Sciences and Technology, Islamabad 44000, Pakistan; 3 Graduate School of Information Science and Technology, Osaka University, 1-2 Yamadaoka, Suita, Osaka 565-0871, Japan; 4 Graduate School of Chemical Sciences and Engineering, Hokkaido University, North 13 West 8, Kita-ku, Sapporo, Hokkaido 060-8628, Japan

## Abstract

Waheed, a former postdoctoral researcher; Gaku, a senior researcher; and Akihiro, a group leader in Okamoto lab succeeded high-quality database construction and discovered a highly stable microbial power-generation mechanism. They talk about how wet electrochemists jumped into the data science field and the potential of data science to explore complex bacteria/electrode interactions.

## Main text

**Figure:**
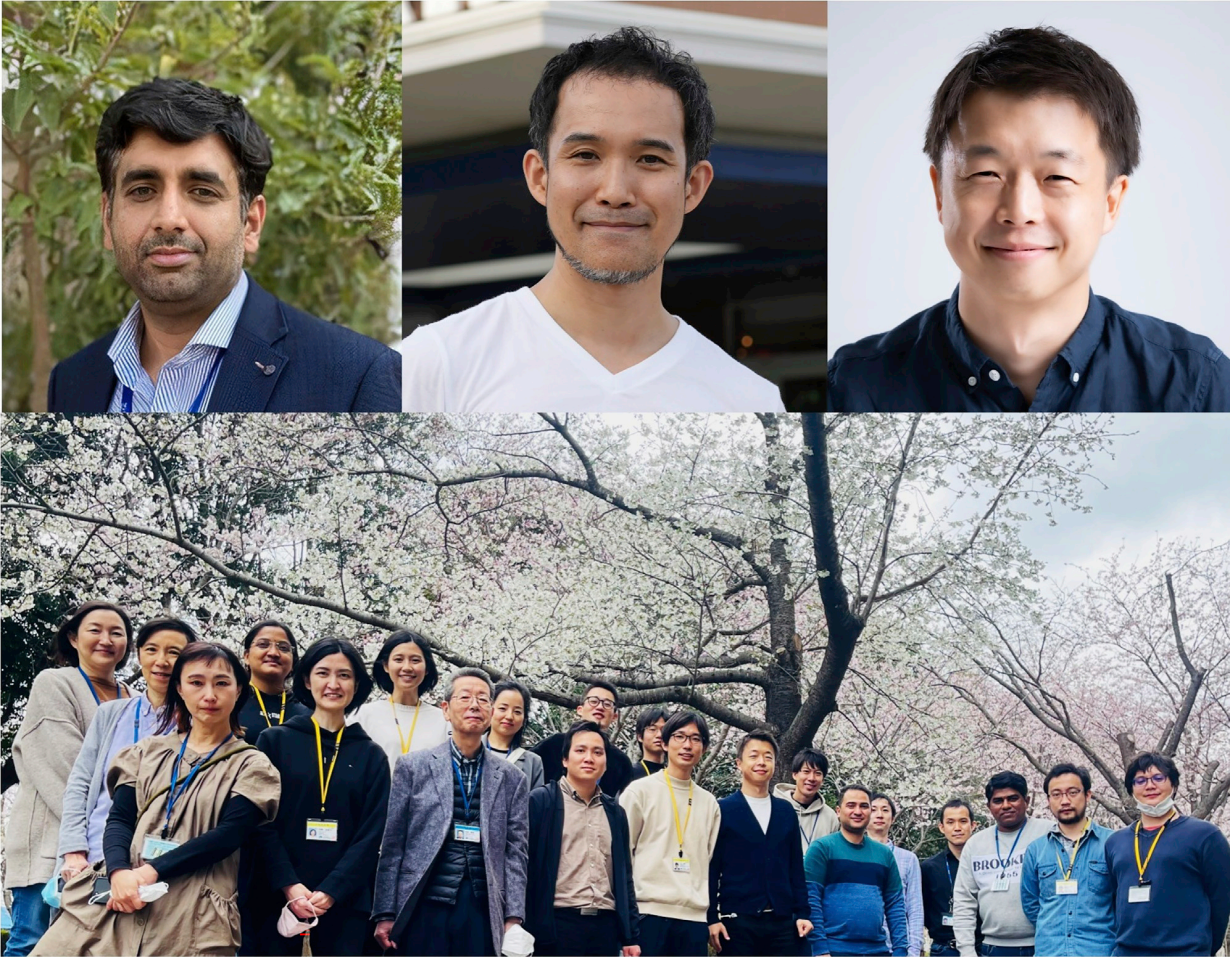
Waheed Miran (left), Gaku Imamura (center), Akihiro Okamoto (right), and members of the Okamoto lab under cherry tree in full bloom (bottom)

### What would you like to share about your background (personal and/or professional)?

**Akihiro Okamoto:** During my undergraduate years at the University of Tokyo, I admired Tadao Ando and aspired to become an architect. My heart soared when I encountered cool architecture, and I loved traveling around confronting the new and the old. However, as I encountered various things in my travels, I became strongly interested in cutting-edge science. I wanted to do basic research in the fields of environment and energy to impact society, so I entered the Department of Applied Chemistry in my second year of undergraduate school, and since then I have spent every waking moment thinking about research. In electrochemistry, my specialty, the experimental setup is not easy, and it is a field where one has to carefully interpret a small amount of data. However, the events that occur at the electrode interface are very complex, and I have always believed that the use of data science should be able to bring innovation to this field.

**Waheed Miran:** After obtaining chemical engineering bachelor’s degree from university of the Punjab, Pakistan, I was trained as a process engineer during my industrial Job at LOTTE Chemical Pakistan, the sole manufacturer of purified terephthalic acid in Pakistan. I did my master’s and PhD in the area of applied bioelectrochemical systems (BESs) in South Korea under the supervision of Dr. Dae Sung Lee, who gave me freedom to work in multidisciplinary domains and investigate the new frontiers in our field. I was further trained in electromicrobiology involving fundamental and curiosity research under the young and dynamic mentorship of Dr. Akihiro Okamoto at National Institute for Materials Science (NIMS), Japan, during my Japan Society for the Promotion of Science (JSPS) fellowship. We have many brainstorming sessions to come up with new dimensions to our research and use of data science for high-throughput systems in BESs is one of them. Currently, I am an assistant professor at School of Chemical and Materials Engineering, National University of Sciences and Technology, Pakistan. Briefly about personal life, I got married at a young age, and now I am a father of a daughter and two sons, spending a happy family life in the capital city of Pakistan, Islamabad.

**Gaku Imamura:** I have been interested in many things since I was a child, and natural science is one of such exciting topics. That is why I wanted to become a researcher. I have been working on olfactory sensors. Olfactory sensors are sensor systems that detect and identify odors and are being studied worldwide. Because I expected data science would be a great tool in olfactory sensors, I started learning data science 6 years ago and utilized it for analyzing the sensor data. Now, I also belong to Osaka University as a guest associate professor and look for students who wish to study and conduct research with us. Moreover, I launched a startup for the odor sensor, Qception Corporation, in 2022 and became the CEO. I want to challenge various things and enjoy my life and career as I’m still interested in many things.

### Where is the team currently based, and how long have you been there?

**AO:** I am originally from a physical chemistry laboratory, and my doctoral thesis was a study on speeding up electron transfer from bacteria to electrodes in microbial fuel cells, which I have been doing for about 15 years since my master’s program on the same topic. I have been working in this interdisciplinary research field, which pursues the interaction between microorganisms and materials (microorganisms and electrodes), initially studying electron transfer mechanisms, but now my research has expanded to various topics including applications and microbiology. Since 2020, I have been working as a group leader of the Electrochemical Nanobiotechnology Group. The current group is largely engaged in data science research based on high-throughput electrochemical measurements,^1^ in addition to microbial electrochemistry. In addition to postdoctoral fellows, we supervise PhD students with backgrounds in chemistry, biotechnologies, and materials science as visiting professors at the Graduate School of Chemical Sciences and Engineering in Hokkaido University.

### Which achievement/discovery in your career are you most proud of?

**AO:** I haven’t actually done any work yet that I can be proud of, but I do have a few goals that I hope to accomplish. I was able to start my research 6 years ago with a budget from AMED with the hypothesis that pathogenic bacteria are power-generating bacteria and that their ability to transfer electrons is related to biofilms and diseases.^2^ However, I have not been able to finish that research yet. It was a research plan with very challenging goals, but I am still convinced that the content is very important. I am also currently engaged in a friendly competition to bring new developments in bioelectrochemistry through the use of data science.^1^ This challenge has also been successful, to some extent. Currently, we are discussing with Dr. Imamura whether it is possible to invent and implement new data scientific methods from the perspective of bioelectrochemistry. I believe that only when we can do this can we say that we have pioneered a new field in which data science and bioelectrochemistry are fused together.

### A lot of data scientists continue their career outside of academia; what is your view on that as a group leader? Do you encourage your students and postdocs to continue their careers in academia and establish their own teams? Are you supportive of careers outside of academia?

**AO:** Currently, one of the postdoctoral researchers in my group is using data science to advance his research. He is working on a project to build a database using the high-throughput electrochemical measurement system reported here and analyze it using data science. We believe that people like him who can do both experiments and data science will be very valuable in academia and industry in the future. This is because if you have the skills to generate high-quality data through experiments, you will be able to take full advantage of the benefits of data science in your research and development. In addition to basic research, the achievement of various more practical objectives, such as product development in industry, will be greatly accelerated. I believe that producing more people like him in the next few years will be our way of repaying our debt to academia and industry, and I feel a strong sense of mission.

### Looking back, what advice would you have given yourself at the start of the career? Is there anything you would have done differently?

**AO:** We never dreamed that we would actually be able to develop a high-throughput electrochemical measurement system as reported in this paper. It is common knowledge that electrochemical devices have large, thick cables stretching across them, and everyone thinks it is insane to arrange 96 of them, much less 1,920, and design more complicated systems that incorporate electrode switching mechanisms. However, coming to the NIMS and meeting professionals in the field of electronic devices made it possible for me to realize such a device. If I could go back 10 years, I would tell myself high-throughput electrochemical systems are feasible and the phone numbers of the professionals.

### As the first author of this paper, what drew you to your current team and topic?

**WM**: Joining Dr. Okamoto’s team at NIMS was an interesting life event for me. Back in 2016, Dr. Okamoto’s group visited Busan, South Korea, for the third AP-ISMET conference. When that conference took place, I was a PhD student in Daegu, South Korea, and also attended this conference. I also participated in the AP-ISMET workshop for newcomers to the subject that was held at the conclusion of the conference. Dr. Okamoto spoke about several electrochemical techniques for studying extracellular electron transport mechanism as one of the workshop presenters, and I found his session really interesting. Later, I contacted him for an internship at his research institute, NIMS, and after few discussions we finalized the internship plan for almost 3 months. There, I studied mechanism of extracellular electron transfer in sulfate reducing bacteria along with a PhD student, and later we also published that work. The discussions during my stay with Dr. Okamoto’s group result in a research proposal on controlling biocorrosion due to electroactive bacteria, which help me in getting a position of NIMS postdoc in his lab. After 1.5 years at NIMS, our new research proposal about studying control strategies for electroactive pathogen and electroactive biocorrosion bacteria was accepted for my JSPS postdoctoral fellowship. During JSPS, we developed our indigenous high-throughput system with high reproducibility for time current profiles from electroactive bacteria. Coupling this system with data science led us to discover new mechanism and optimization of operating conditions. We believe it is a breakthrough in an extremely essential area and will be a game changer in our field.

### Was there a particular element (paper, collaboration, talk/conference, key experiment, idea, result) that motivated you to start/participate in this project?

**WM:** After the development of our original high-throughput electrochemical measurement system and fixing all the startup and software issues, it was possible to accelerate the electrochemical measurement hundreds of folds faster than conventional technology to construct a highly reproducible database. Initially we were checking the reproducibility with *Shewanella oneidensis* MR-1, one of the most studied electroactive organisms regarding extracellular electron transfer. After achieving very high reproducibility data in comparison to studies in literature, it motivated us to include data science to dig deep and elucidate mechanism in more diverse conditions, i.e., uninvestigated region of electrode potential and mediators.

### We are curious about your next the project, what’s next for you?

**WM:** We want to develop metabolic activity sensor technologies for antibiotic drug discovery. Our high-throughput system can really expedite the screening process for finding effective drugs against electroactive pathogens. I want to extend the research for finding new electroactive pathogens and discovery of new natural products with antimicrobial activities.

### What is the definition of data science in your opinion? What is a data scientist? Do you self-identify as one?

**WM:** In my opinion, data science is a way of analyzing complex interactions in different processes that are sometimes even beyond our imagination. Data scientist is the one that use huge amounts of data analyzed by using cutting-edge tools and methods to uncover previously unnoticed patterns. I see myself as a novice in this field who is eager to go further into and learn about the emerging field of data science.

**GI:** A data scientist should be a person who develops new informatic techniques such as theories, algorithms, and frameworks. In that sense, I am not a data scientist because I just apply existing data scientific techniques to developing olfactory sensors. But I strongly believe that applying data scientific techniques to a specific research field requires a deep expertise and knowledge of the field. So, I think that the definition of data science should include appropriately utilizing data scientific tools for a specific field.

### What motivated you to become a (data) researcher? Is there anyone/anything that helped guide you on your path?

**WM:** Curiosity is what drives me to pursue a career in research. I am constantly curious about undiscovered facts, and I investigate them thoroughly to learn the true science involved. One of my managers in my industrial job, my PhD advisor, and my postdoc supervisor all have a significant influence on how I develop as a researcher and critical thinker. The previous few years have been incredibly fruitful in helping me shape up my career as a researcher and defining my path, all thanks to a terrific team at NIMS who excelled in the different dimensions in the field of electromicrobiology.

**GI:** I was interested in fundamental science and working on synthesizing and analyzing new materials when I was a student. After I received my PhD degree in chemistry in 2013, I wished to expand the scope of my study and started working on olfactory sensors, in which various research fields are involved. At this time, I recognized the importance of data science in developing olfactory sensors. In the project of developing olfactory sensors, I met a distinguished data scientist Prof. Washio from Osaka University and discussed over and over about how to analyze sensor data. If it had not been for him, I should have never learned data science.

### Why did you decide to publish in *Patterns*?

**WM:** We were motivated to submit our work to *Patterns* since the journal has established a strong reputation in a short span of time for disseminating original research that is cutting edge and covers the whole gamut of data science.

**GI:** I felt this study was interdisciplinary and needed to be published in a journal that welcomes nonconventional research.

### Which of the current trends in data science seem most interesting to you? In your opinion, what are the most pressing questions for the data science community?

**WM:** It is different from my core field, but as I am always interested in world politics and how government functions, using data science for public budgeting is an interesting phenomenon taking shape and expected to lessen the human intervention in future in one of the most significant internal responsibilities of government. In this regard, one of the newest developments is the use of automated machine learning that will revolutionize the data science.

The most important issue facing the data science community, in my opinion, is data quality. Making a thorough strategy from the start is the answer to this issue. Concentrate on developing a solid foundational system, as a better data structure will undoubtedly make it simpler to analyze data.

### How do you keep up to date with advances in both data science techniques and in your field/domain?

**AO:** I myself am still learning about data science and do not have a good understanding of the latest research situation. So basically, I am developing a research plan by collaborating with data scientists to match our needs and what data science can do. Also, there is still very little research combining data science and bioelectrochemistry. So, until now, we have been asking Dr. Imamura to help us in how we analyze high-throughput data and extract scientifically meaningful information. From now on, however, we hope to gain a deeper understanding of how research is conducted in the field of data science and develop universal data science analysis methods. For this purpose, I believe it is necessary to participate in academic conferences and have more active discussions with data scientists within the NIMS.

### What is the role of data science in your domain/field? What advancements do you expect in data science in this field over the next 2–3 years?

**AO:** Until now, data science has been used to extract scientific insights from high-throughput-measured electrochemical databases, as mentioned earlier. However, I believe that in the future, it will be important to develop data scientific methods rooted in the measurement system of bioelectrochemistry. For example, electrochemical measurements can be linked to information from spectroscopy and microscopy, and I expect that higher-resolution scientific information can be obtained by using data science. In this way, I believe that we will see new ways and needs to utilize data science by linking it to high-throughput experimental systems, and I expect to see an increase in similar cases over the next few years. We hope to advance our research well so that we can be the first to take the lead in this process.

**WM:** Data science can really help in our field for fundamental understanding of BESs that will enable complex BES models to improve the performance of practical systems. In our field, we anticipate that the combination of data science and high-throughput electrochemistry will greatly accelerate breakthrough for carbon-neutral electrochemical technologies. Over the next 2–3 years, I can expect our high-throughput systems coupled with data science models to be available to different stakeholders and to be used practically for fields such as drug testing and antimicrobial activities.

**GI:** Data science is currently a novel and powerful tool in the field of microbial electrochemistry and olfactory sensors, but it would become a common tool in the near future after the accumulation of many reports that utilize data science in these fields.

### What is your advice for future data scientists, or what attributes do you think make a data scientist successful?

**AO:** After all, isn’t it important in this field in the future not only to use existing databases but also to work together with experimental researchers to create databases and design experiments? At NIMS, we have materials scientists, researchers who evaluate materials by electrochemistry, and data scientists, and we have an environment where materials exploration research using informatics can proceed. Here, we also need more technology to create materials in a high-throughput manner, and we need to solve various challenges such as how to create databases and how to link multimodal data to search for promising materials. I expect that progress in each technology will be made in how to accomplish these tasks under these tough constraints.

**WM:** There are many attributes required to make a data scientist successful such as curiosity, critical thinking, analytical skills, and persistence, but since data science is an interdisciplinary field that integrates programming, design, mathematics, statistics, and scientific communication, it can have countless applications and may be used to solve issues in all kinds of industries. Therefore, flexibility and adaptability are key qualities for every data scientist to possess.

**GI:** Although developing new data scientific technologies itself is important for data scientists, I believe how to apply data science to each specific field would create values in the future. Data scientists who have specialty not only in data science but also in a field would be highly recognized.
